# Performance of optimized McRAPD in identification of 9 yeast species frequently isolated from patient samples: potential for automation

**DOI:** 10.1186/1471-2180-9-234

**Published:** 2009-11-10

**Authors:** Jitka Trtkova, Petr Pavlicek, Lenka Ruskova, Petr Hamal, Dagmar Koukalova, Vladislav Raclavsky

**Affiliations:** 1 Department of Microbiology, Faculty of Medicine & Dentistry, Palacky University and University Hospital Olomouc, Hnevotinska 3, 775 15 Olomouc, Czech Republic

## Abstract

**Background:**

Rapid, easy, economical and accurate species identification of yeasts isolated from clinical samples remains an important challenge for routine microbiological laboratories, because susceptibility to antifungal agents, probability to develop resistance and ability to cause disease vary in different species. To overcome the drawbacks of the currently available techniques we have recently proposed an innovative approach to yeast species identification based on RAPD genotyping and termed McRAPD (Melting curve of RAPD). Here we have evaluated its performance on a broader spectrum of clinically relevant yeast species and also examined the potential of automated and semi-automated interpretation of McRAPD data for yeast species identification.

**Results:**

A simple fully automated algorithm based on normalized melting data identified 80% of the isolates correctly. When this algorithm was supplemented by semi-automated matching of decisive peaks in first derivative plots, 87% of the isolates were identified correctly. However, a computer-aided visual matching of derivative plots showed the best performance with average 98.3% of the accurately identified isolates, almost matching the 99.4% performance of traditional RAPD fingerprinting.

**Conclusion:**

Since McRAPD technique omits gel electrophoresis and can be performed in a rapid, economical and convenient way, we believe that it can find its place in routine identification of medically important yeasts in advanced diagnostic laboratories that are able to adopt this technique. It can also serve as a broad-range high-throughput technique for epidemiological surveillance.

## Background

Over the past decades, patients have increasingly been colonized and infected with a variety of yeast species mainly due to immunocompromising conditions as well as due to increased use of invasive techniques and devices (for reviews see [1,2]). Therefore, clinical microbiology laboratories face an important challenge of rapid detection of pathogenic yeasts. However, accurate species identification is very much demanded in addition to mere detection, because susceptibility to antifungal agents, probability of resistance development and ability to cause disease vary in different species [3]. Although there are several rapid diagnostic procedures available based mainly on PCR amplification of yeast DNA that have been developed to facilitate diagnosis, conventional cultivation techniques followed by identification of pure culture still dominate the field. A profound change can hardly be expected in the foreseeable future except for rapid detection of selected yeasts species in specific types of samples, blood in particular. This is mainly because only the identification techniques based on pure culture examination are able to identify the whole spectrum of potentially pathogenic yeast species reliably. Also, only cultivation techniques make antifungal susceptibility testing and strain typing for epidemiological purposes possible. However, diagnostic laboratories and clinicians can hardly be satisfied with the potential of routinely available identification techniques in this field because these are typically either (i) economical and easy to perform but time-consuming, or (ii) rapid but costly and/or requiring special equipment or expertise. For reviews on phenotyping- and genotyping-based systems see [4,5].

We have recently proposed an innovative technique termed McRAPD (Melting curve of Random Amplified Polymorphic DNA) which has the potential to provide rapid and accurate pathogenic yeast identification grown in pure culture in an easy and economical way [6]. Here we have evaluated the performance of optimized McRAPD on a broader spectrum of yeast species frequently isolated from clinical samples and also examined the potential of automated and semi-automated interpretation of McRAPD data for identification purposes. We believe that because of its advantages over conventional phenotypic approaches and its competitive costs, McRAPD can find its place in routine identification of medically important yeasts.

## Results

### Crude colony lysates perform satisfactorily in McRAPD

**Figure 1:**
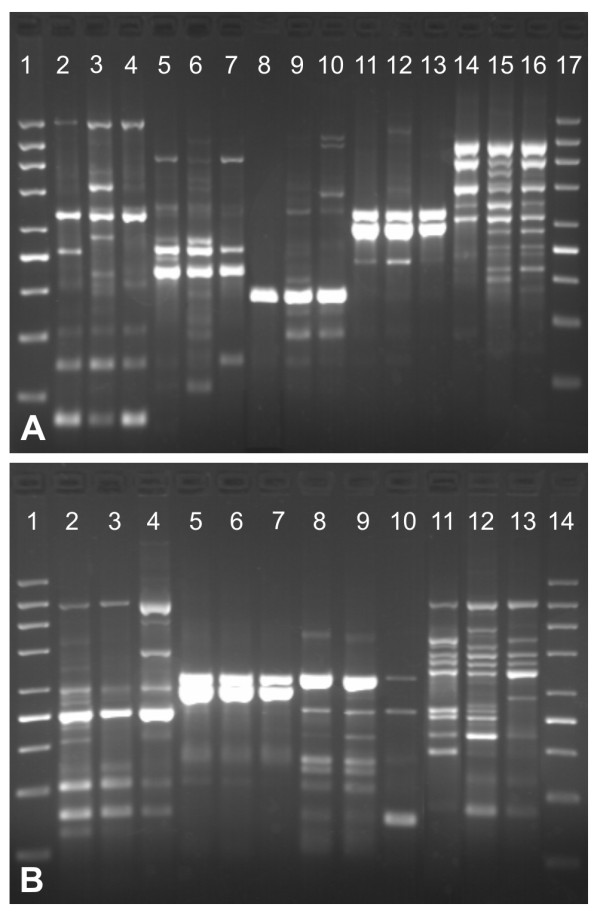
**Results of optimization of the amount of crude colony lysates added into reaction mixture**. Lanes are arranged in triplicates where each triplicate of lanes represents results obtained with the same strain. Individual lanes within each triplicate represent variable amount of crude colony lysate added into the reaction mixture, namely 0.5, 1, and 2 μl in the order from left to right. Part (A), lane 1 and 17: molecular weight marker 200-1500 (Top-Bio, Prague, Czech Republic), lanes 2-4: *C. albicans *ATCC 76615; lanes 5-7: *C. krusei *I1-CAKR-24; lanes 8-10: *C. tropicalis *I3-CATR9-37; lanes 11-13: *C. lusitaniae *I1-CALU-33; lanes 14-16: *C. parapsilosis *CBS 604; part (B), lane 1 and 14: molecular weight marker 200-1500 (Top-Bio, Prague, Czech Republic), lanes 2-4: *C. pelliculosa *I3-CAPE3-10; lanes 5-7: *C. guilliermondii *I1-CAGU2-20; lanes 8-10: *S. cerevisiae *I3-SACE3-37; lanes 11-13: *C. glabrata *I1-CAGL-32.

**Figure 2:**
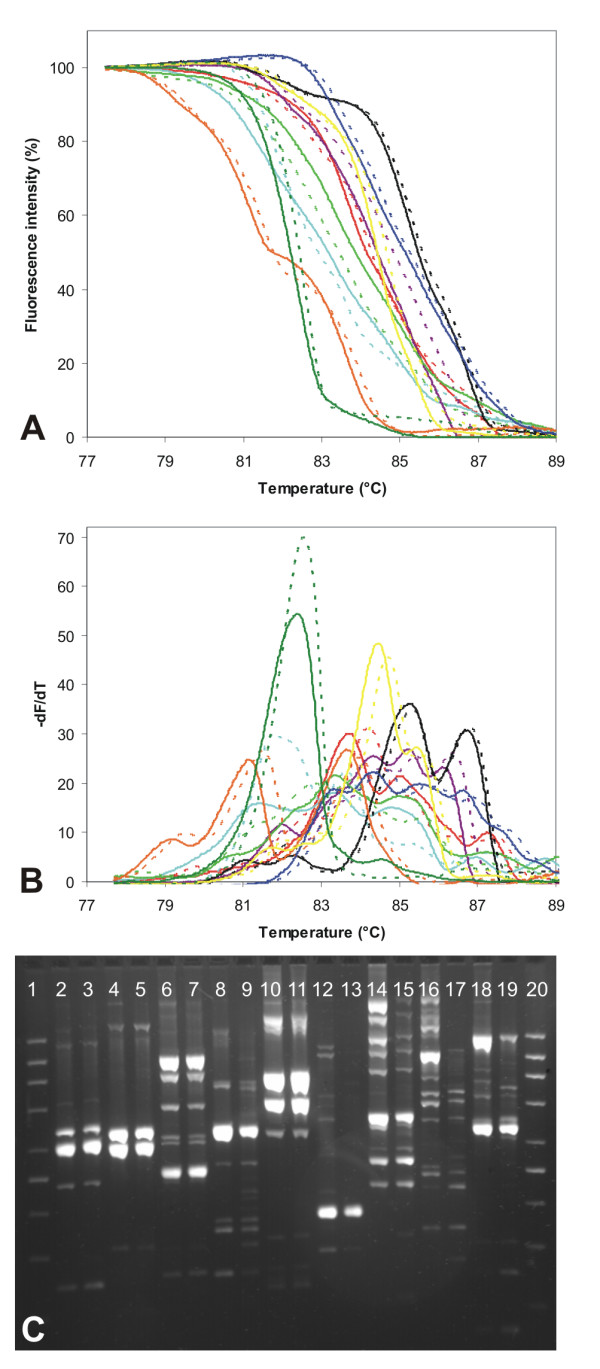
**Comparison of McRAPD results obtained with DNA extracted using the commercial kit YeaStar Genomic DNA Kit (*Zymo Research, Orange, CA, USA*) and using the technique of crude colony lysates**. Selected strains were subjected to DNA extraction in parallel and the DNA was used for McRAPD resulting in duplicates of melting curves and duplicates of agarose gel fingerprints. In each duplicate solid lines in plots and left lanes in gel represent results obtained with the DNA extracted using the commercial kit, whereas dotted lines and right lanes represent results obtained with 1 μl of crude colony lysate. Part (A): normalized melting curves, part (B) derivative curves, part (C) fingerprints obtained with agarose gel electrophoresis, lane 1 and 20 molecular weight marker 200-1500 (Top-Bio, Prague, Czech Republic). Lane 2, 3 and black line *C. lusitaniae *I1-CALU-33, lane 4, 5 and violet line *C. guilliermondii *I1-CAGU2-20, lane 6, 7 and blue line *C. pelliculosa *I3-CAPE3-10, lane 8, 9 and yellow line *S. cerevisiae *I3-SACE3-37, lane 10, 11 and orange line *C. metapsilosis *I1-CAME7-11, lane 12,13 and dark green line *C. tropicalis *I3-CATR9-22, lane 14, 15 and light green line *C. krusei *I1-CAKR-24, lane 16, 17 and turquoise line *C. glabrata *I1-CAGL-39, lane 18, 19 and red line *C. albicans *ATCC 76615.

To achieve rapid and economical performance of the McRAPD identification approach, we used the simplified DNA extraction technique described by Steffan et al. [7]. However, since the recommended 0.3 μl volume of crude colony lysates added into McRAPD reaction mixture did not always provide satisfactory results with all the species included in our study, we first optimized this volume. Results of optimization are summarized in Figure 1. Apparently, the volume of crude colony lysates added into the reaction mixture had no or almost no influence on the banding pattern in most of the species, whereas there were marked differences in others (namely *S. cerevisiae *and *C. glabrata*). Based on these results, the amount of 1 μl was evaluated as the best, because lower volumes did not always guarantee reliable amplification, whereas in higher volumes it cannot be excluded that too much PCR inhibitors may enter the reaction mixture. For comparison and reference, the commercial kit YeaStar Genomic DNA Kit (Zymo Research, Orange, California, USA) was used in parallel with 1 μl of crude colony lysates. Results of this comparison represented by melting curves and banding patterns are summarized in Figure 2. When comparing the initial relative fluorescence of amplified samples, the use of DNA extracted by the commercial kit resulted in higher values on average, indicating higher yields. In 8 of the 9 species studied, no marked differences in melting curves based on kit versus crude lysates were observed, although some minor differences in the relative intensity of individual bands occurred in some of the species. Only 1 of the 9 species, namely *C. glabrata*, showed both markedly different banding patterns and melting curves, indicating that the performance of McRAPD with colony lysate was suboptimal in this case compared to the commercial kit. Our experience in routine experiments shows that the initial relative fluorescence intensity of a McRAPD sample after amplification should exceed the relative value of 15 at the standard 30% LED power as adjusted in melting protocol by user. When a sample does not meet this condition, repeating the assay including DNA extraction is strongly recommended for reliable results.

**Figure 3:**
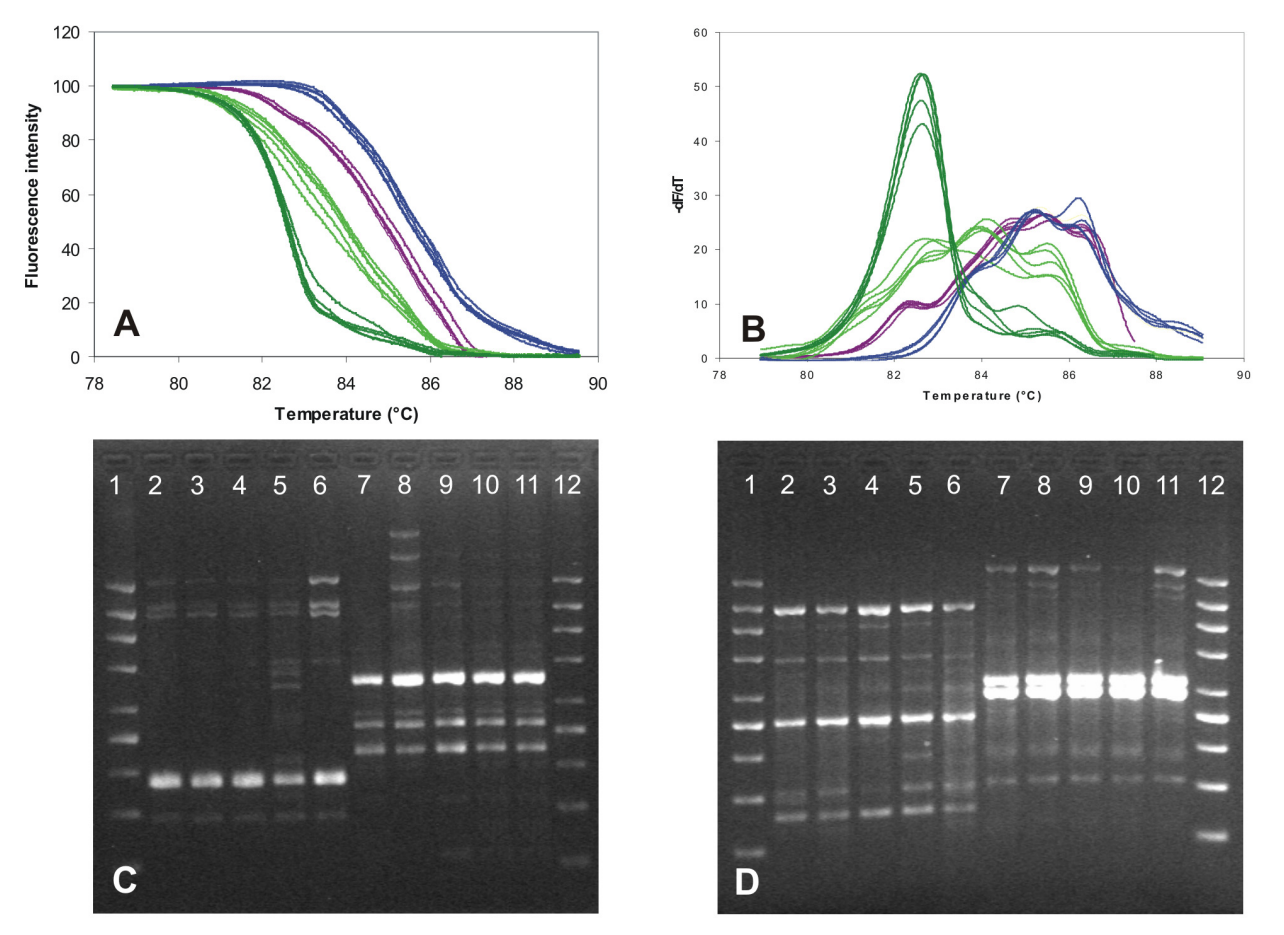
**Reproducibility of McRAPD with crude colony lysates sampled from different colonies at different timepoints**. DNA extraction was performed in 4 different yeast species, each represented by one strain, where 5 colonies were subcultured for different time periods in each strain. Part (A) normalized melting curves, part (B) derivative curves, part (C) and (D) fingerprints after agarose gel electrophoresis with the 200-1500 molecular weight marker (Top-Bio, Prague, Czech Republic) in lanes 1 and 12. Dark green lines and lanes 2-6 in part (C) *C. tropicalis *I3-CATR9-17; light green lines and lanes 7-11 in part (C) *C. krusei *I1-CAKR-06; violet lines and lanes 2-6 in part (D) *C. pelliculosa *I3-CAPE3-04; and blue lines and lanes 7-11 in part (D) *C. guilliermondii *I1-CAGU-22.

In addition, reproducibility of the simplified DNA extraction based on crude colony lysates was tested. DNA was extracted from 4 different yeast species, each represented by one strain, where 5 colonies were grown for different time periods in each strain and used for extraction. Sampling was performed in the interval between 12 and 24 h of colony growth, approximately every 3 h. Freshly prepared lysis buffer was always used for DNA extraction in each of the samples. The results clearly demonstrate that the time-point of colony sampling and different runs of the extraction procedure have little influence on the variability of McRAPD results (Figure 3). Our data show, that crude colony lysates perform satisfactorily in McRAPD. Of course, any DNA extraction technique may fail to provide adequate amplification occasionally and a commercial kit should on average secure better reproducibility compared to the technique of crude colony lysates. As widely accepted, commercial kits should also be generally more robust in hands of less experienced personnel. Our experience showed that accurate reproducible sampling of colonies by trained personnel was rather important to achieve reliable amplification with crude colony lysates. Also, using Zymolyase from different suppliers or even different batches of this enzyme from the same supplier can influence performance of the technique. Thus, the procedure needs to be optimized in each laboratory to achieve balance between the amount of cells added into lysing solution and activity of the Zymolyase. Adding too many cells can result in insufficient cell wall lysis and too high concentration of PCR inhibitors. On the contrary, an overload of Zymolyase can be a source of too large amount of contaminating DNA which can interfere with appropriate McRAPD performance, because the McRAPD approach has the capacity to amplify any DNA sample.

### Inter-run variability is very low, whereas inter-strain differences can be a source of considerable variability of McRAPD data in some species

**Figure 4:**
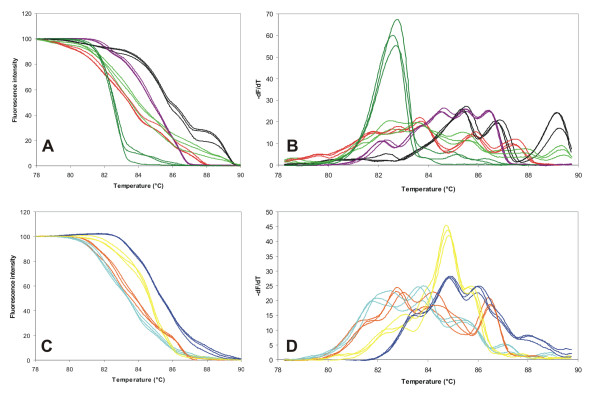
**Short-term stability of crude colony lysates versus reproducibility of McRAPD data as well as evaluation of inter-run variability of McRAPD data**. McRAPD was performed with the same crude colony lysates obtained from 9 strains repeatedly during 3 consecutive days. Parts (A, C) show normalized melting curves, parts (B, D) show derivative curves. Red lines represent *C. albicans *strain I1-CAAL2-38; dark green lines *C. tropicalis *I3-CATR9-13; light green lines *C. krusei *I3-CAKR2-18; violet lines *C. guilliermondii *I1-CAGU2-21; black lines *C. lusitaniae *I1-CALU2-32 (all in parts A and B); turquoise *C. glabrata *I3-CAGL2-15; orange *C. parapsilosis *I1-CAPA7-28; blue *C. pelliculosa *I3-CAPE3-04; and yellow *S. cerevisiae *I1-SACE2-40 (all in parts C and D).

**Figure 5:**
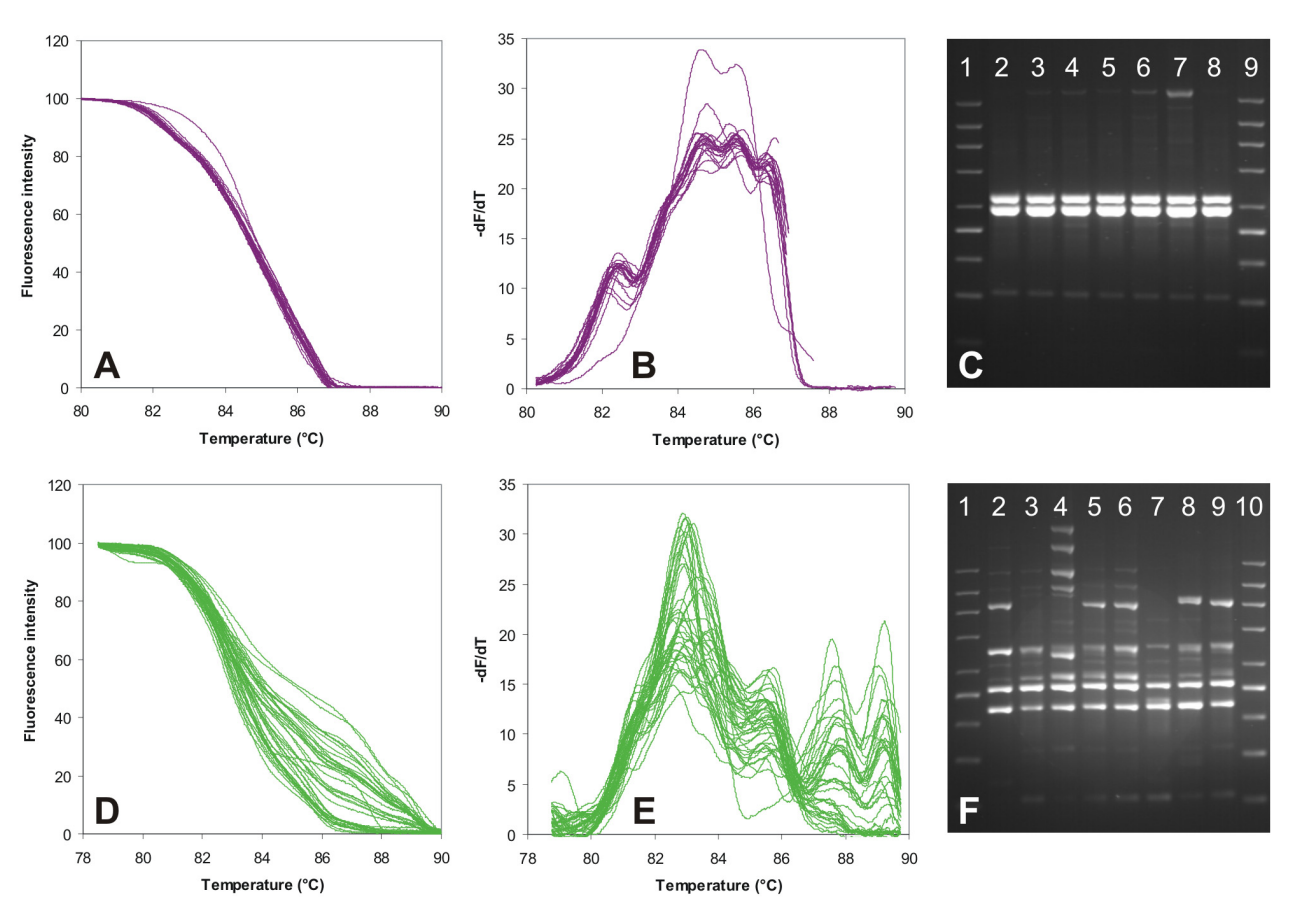
**Interstrain variability of McRAPD data in *C. guilliermondii *(parts A-C; lowest variability in this study) and *C. krusei *(parts D-F; highest in this study)**. Parts (A, D) show normalized melting curves, parts (B, E) show derivative curves, parts (C, F) show fingerprints after agarose gel electrophoresis with the 200-1500 molecular weight marker (Top-Bio, Prague, Czech Republic) in lanes 1 and 9 and 10, respectively. All strains of the respective species included in the study are plotted, whereas only fingerprints of selected strains are demonstrated, namely lane 2: I1-CAGU2-35, lane 3: I1-CAGU2-34, lane 4: I1-CAGU2-33, lane 5: I1-CAGU2-32, lane 6: I1-CAGU2-31, lane 7: I1-CAGU2-30, lane 8: I1-CAGU2-29 (all *C. guilliermondii*)in part (C); lane 2: I3-CAKR2-33, lane 3: I3-CAKR2-32, lane 4: I3-CAKR2-31, lane 5: I3-CAKR2-30, lane 6: I3-CAKR2-29, lane 7: I3-CAKR2-28, lane 8: I3-CAKR2-27, lane 9: I3-CAKR2-26 (all *C. krusei*) in part (F).

We have repeated McRAPD amplification with the same crude colony lysates during 3 consecutive days to test for the short-term stability of DNA in these lysates and to evaluate the inter-run variability of McRAPD data. Results are demonstrated in Figure 4; no marked differences were observed indicating that the McRAPD technique itself performed highly reproducibly. We have also tested the influence of short-term storage of crude colony lysates at -20°C on proper performance and reproducibility of McRAPD and have not observed any marked variability (data not shown). On the contrary, considerable interstrain differences were observed when performing McRAPD in some species, whereas rather uniform data were observed in other species. The lowest interstrain variability was observed in *C. guilliermondii*, whereas the highest was observed in *C. krusei *(Figure 5). It can be supposed, that the species showing typically simple fingerprints with just one or only a few intense bands and almost no interstrain variability should produce less variable melting curves, whereas those showing complex and variable fingerprints should produce rather variable melting curves. This assumption is in good agreement with the fingerprinting patterns of selected strains of *C. guilliermondii *and *C. krusei*, as demonstrated in Figure 5C, F. This figure also illustrates that the uniformly present shorter RAPD products (around 500 bp) are reflected in the uniform first portion of the melting domain in *C. krusei *(78-82.5°C), whereas those variably present longer RAPD products (> 900 bp) are reflected in the variable second portion of the domain (82.5-90°C, compare Figure 5D-F). Marked differences in interstrain variability in different species observed by us are not surprising, because previous studies showed rather different degrees of genotypic variability in different yeast species [8-10]. Thus, although our McRAPD protocol was previously optimised empirically to achieve the highest uniformity of data within each species, some of the species studied have too many variables in their genotypes to provide uniform data with our protocol. Although this drawback can potentially hinder simple species identification, it might be compensated by the fact that detection of outstanding interstrain differences could provide valuable genotyping data along with identification in some of the species studied.

### Different genotypes can be recognized within species based on McRAPD data

**Figure 6:**
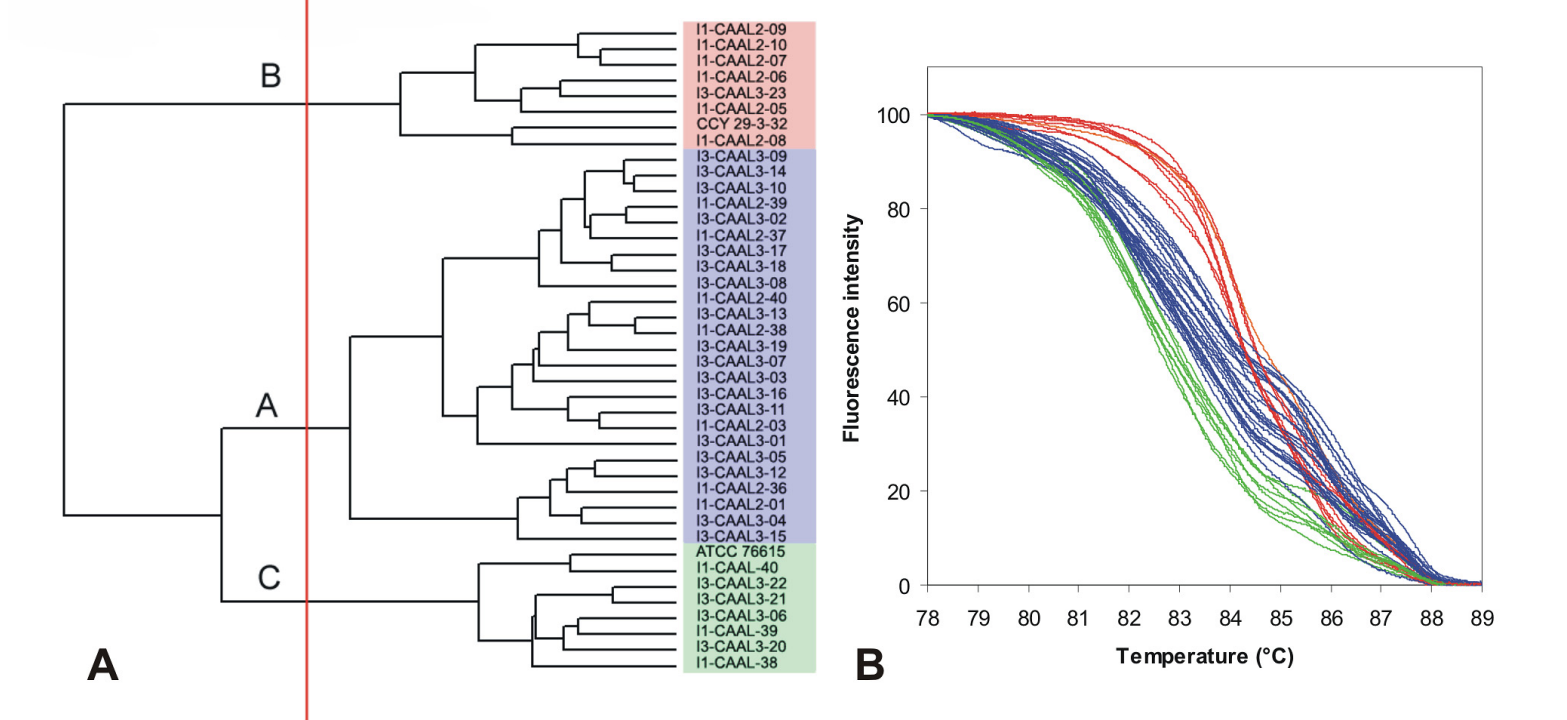
**UPGMA clustering of *C. albicans *strains based on normalized McRAPD data**. Clustering with empirically defined genotypes is demonstrated in part (A) and corresponding normalized melting curves are shown in part (B). All strains of the respective species included in the study are clustered and plotted; strains belonging to a specific genotype are highlighted by specific ground tint color in the dendrogram corresponding with the same color of curves in accompanying normalized melting curve plot and derivative plots.

**Figure 7:**
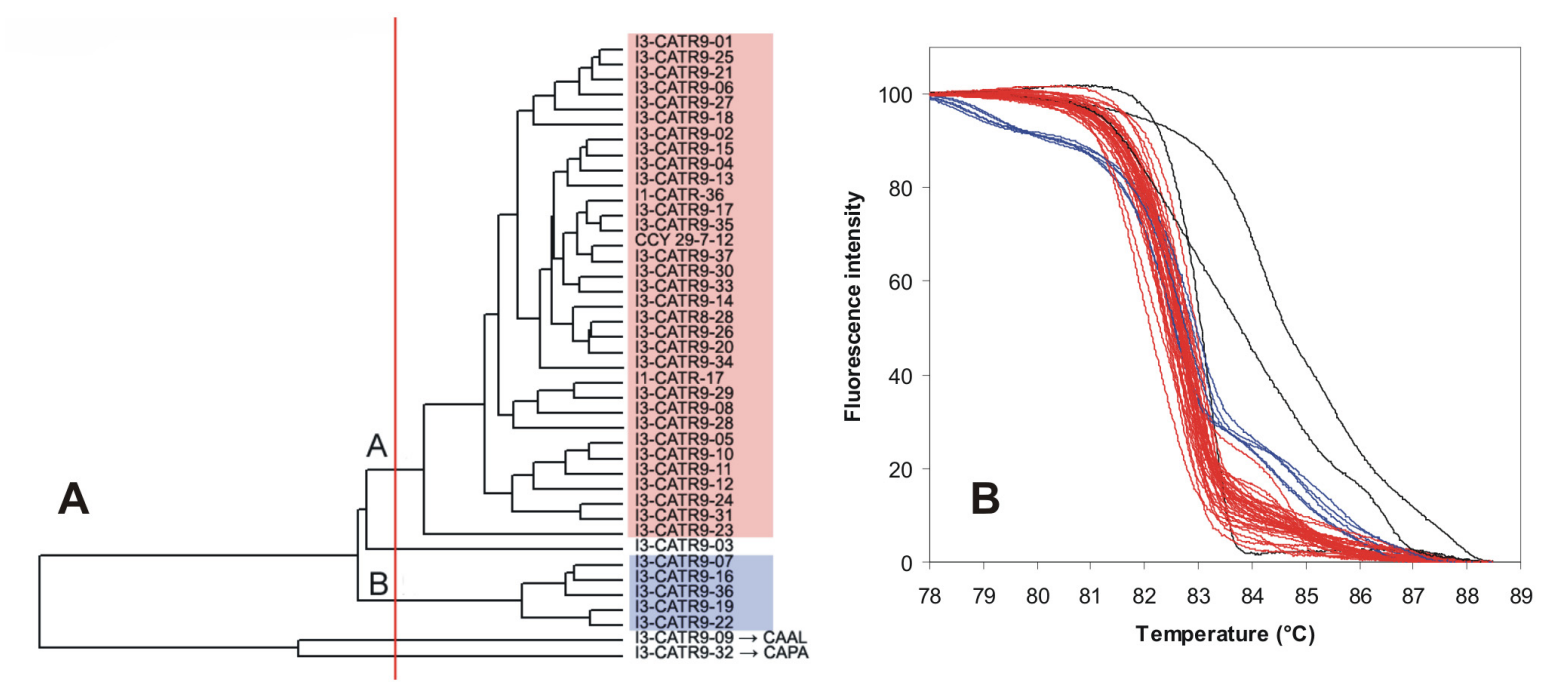
**UPGMA clustering of *C. tropicalis *strains based on normalized McRAPD data**. Clustering with empirically defined genotypes is demonstrated in part (A) and corresponding normalized melting curves are shown in part (B). All strains of the respective species included in the study are clustered and plotted; strains belonging to a specific genotype are highlighted by specific ground tint color in the dendrogram corresponding with the same color of curves in accompanying normalized melting curve plot and derivative plots. Three strains not assigned to a specific genotype are not color-coded in dendrogram and their melting curves are plotted in black. Two of these strains were later re-identified as *C. albicans *and *C. parapsilosis*.

**Figure 8:**
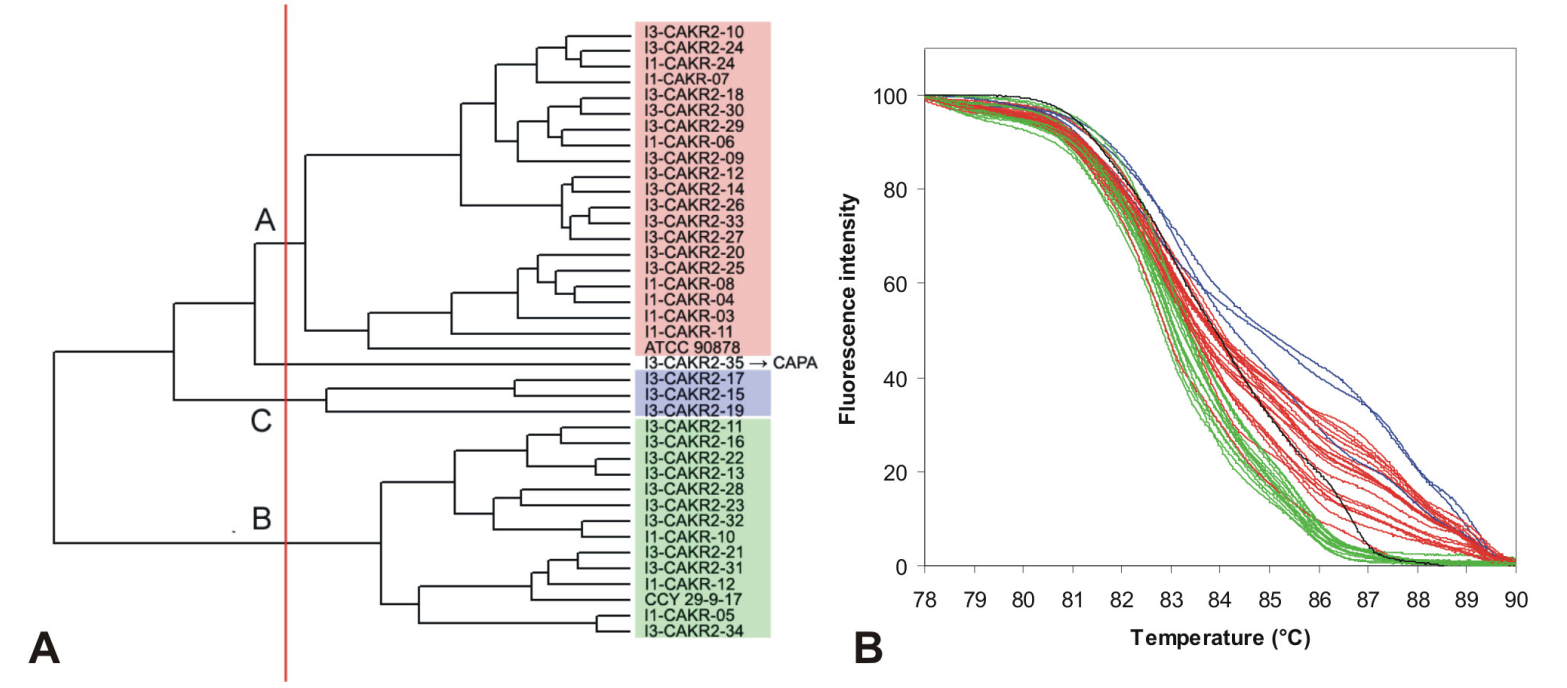
**UPGMA clustering of *C. krusei *strains based on normalized McRAPD data**. Clustering with empirically defined genotypes is demonstrated in part (A) and corresponding normalized melting curves are shown in part (B). All strains of the respective species included in the study are clustered and plotted; strains belonging to a specific genotype are highlighted by specific ground tint color in the dendrogram corresponding with the same color of curves in accompanying normalized melting curve plot and derivative plots. One strain not assigned to a specific genotype is not color-coded in dendrogram and its melting curve is plotted in black. This strain was later re-identified as *C. parapsilosis*.

**Figure 9:**
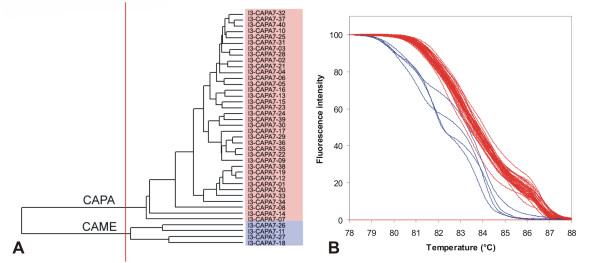
**UPGMA clustering of *C. parapsilosis *strains based on normalized McRAPD data**. Clustering with empirically defined genotypes is demonstrated in part (A) and corresponding normalized melting curves are shown in part (B). All strains of the respective species included in the study are clustered and plotted; strains belonging to a specific genotype are highlighted by specific ground tint color in the dendrogram corresponding with the same color of curves in accompanying normalized melting curve plot and derivative plots.

**Figure 10:**
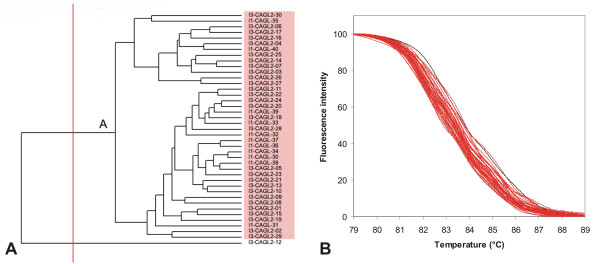
**UPGMA clustering of *C. glabrata *strains based on normalized McRAPD data**. Clustering with empirically defined genotypes is demonstrated in part (A) and corresponding normalized melting curves are shown in part (B). All strains of the respective species included in the study are clustered and plotted; strains belonging to a specific genotype are highlighted by specific ground tint color in the dendrogram corresponding with the same color of curves in accompanying normalized melting curve plot and derivative plots. One strain not assigned to a specific genotype is not color-coded in dendrogram and its melting curve is plotted in black.

**Figure 11:**
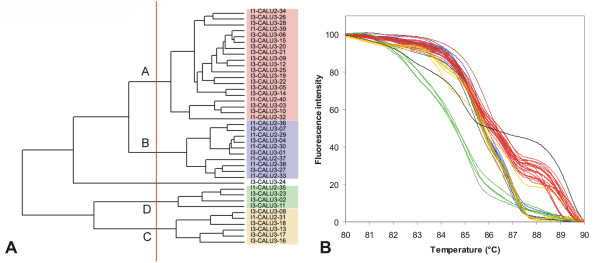
**UPGMA clustering of *C. lusitaniae *strains based on normalized McRAPD data**. Clustering with empirically defined genotypes is demonstrated in part (A) and corresponding normalized melting curves are shown in part (B). All strains of the respective species included in the study are clustered and plotted; strains belonging to a specific genotype are highlighted by specific ground tint color in the dendrogram corresponding with the same color of curves in accompanying normalized melting curve plot and derivative plots. One strain not assigned to a specific genotype is not color-coded in dendrogram and its melting curve is plotted in black.

**Figure 12:**
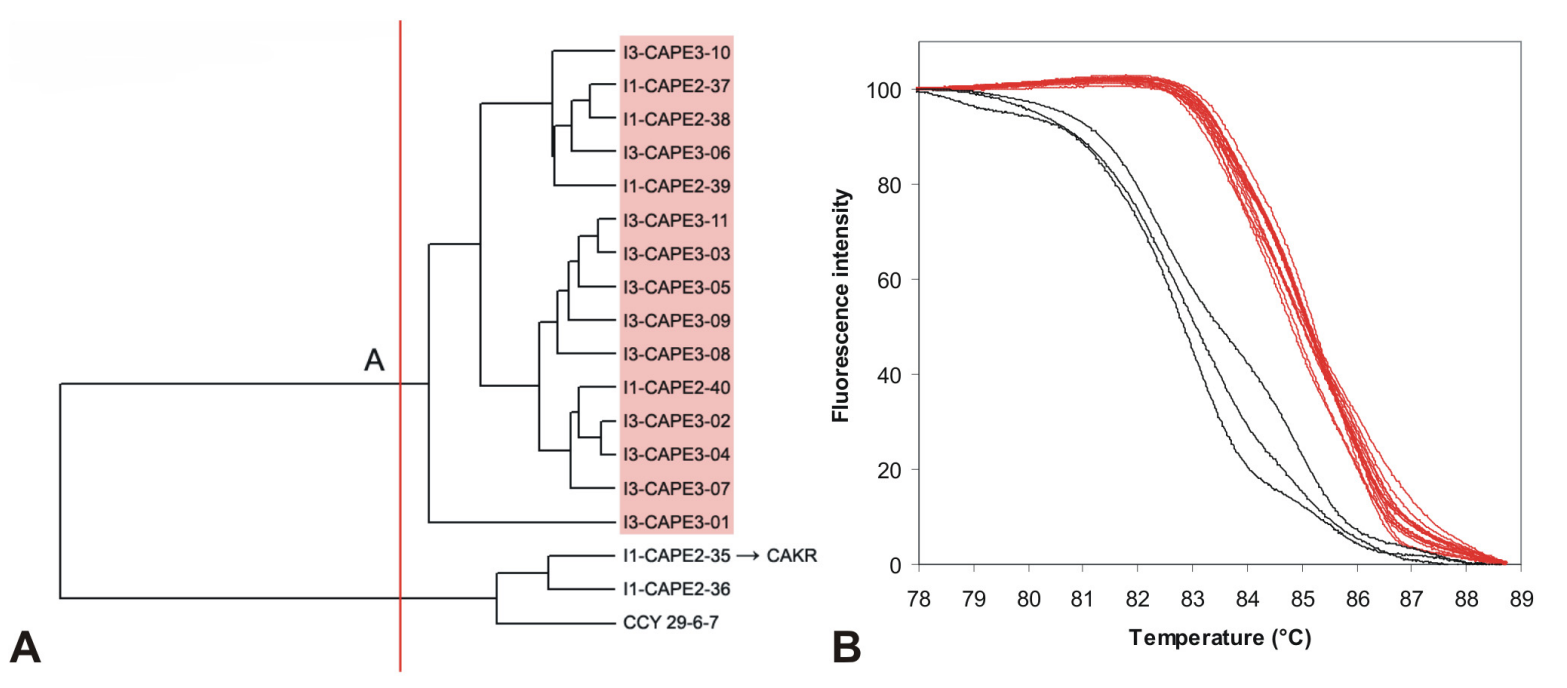
**UPGMA clustering of *C. pelliculosa *strains based on normalized McRAPD data**. Clustering with empirically defined genotypes is demonstrated in part (A) and corresponding normalized melting curves are shown in part (B). All strains of the respective species included in the study are clustered and plotted; strains belonging to a specific genotype are highlighted by specific ground tint color in the dendrogram corresponding with the same color of curves in accompanying normalized melting curve plot and derivative plots. Three strains not assigned to a specific genotype are not color-coded in dendrogram and their melting curves are plotted in black. One of these strains was later re-identified as *C. krusei*.

**Figure 13:**
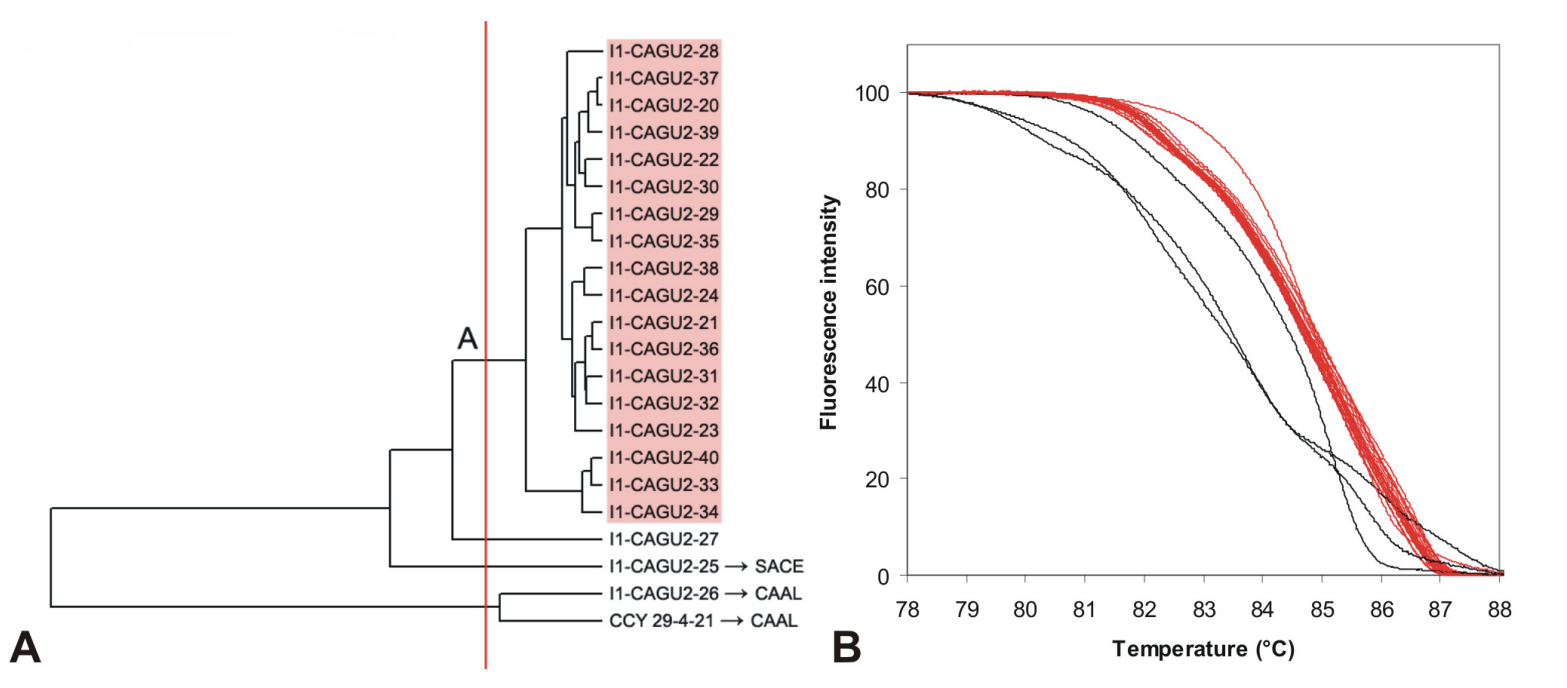
**UPGMA clustering of *C. guilliermondii *strains based on normalized McRAPD data**. Clustering with empirically defined genotypes is demonstrated in part (A) and corresponding normalized melting curves are shown in part (B). All strains of the respective species included in the study are clustered and plotted; strains belonging to a specific genotype are highlighted by specific ground tint color in the dendrogram corresponding with the same color of curves in accompanying normalized melting curve plot and derivative plots. Four strains not assigned to a specific genotype are not color-coded in dendrogram and their melting curves are plotted in black. Two of these strains were later re-identified as *C. albicans *and another one as *S. cerevisiae*.

**Figure 14:**
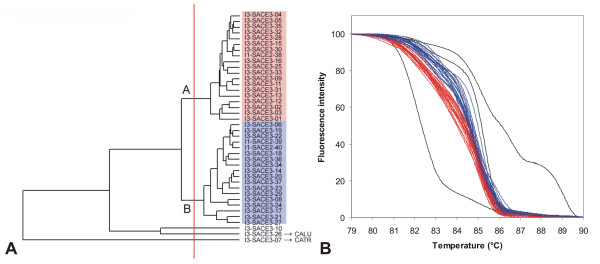
**UPGMA clustering of *Saccharomyces cerevisiae *strains based on normalized McRAPD data**. Clustering with empirically defined genotypes is demonstrated in part (A) and corresponding normalized melting curves are shown in part (B). All strains of the respective species included in the study are clustered and plotted; strains belonging to a specific genotype are highlighted by specific ground tint color in the dendrogram corresponding with the same color of curves in accompanying normalized melting curve plot and derivative plots. Three strains not assigned to a specific genotype are not color-coded in dendrogram and their melting curves are plotted in black. Two of these strains were later re-identified as *C. lusitaniae *and *C. tropicalis*.

**Figure 15:**
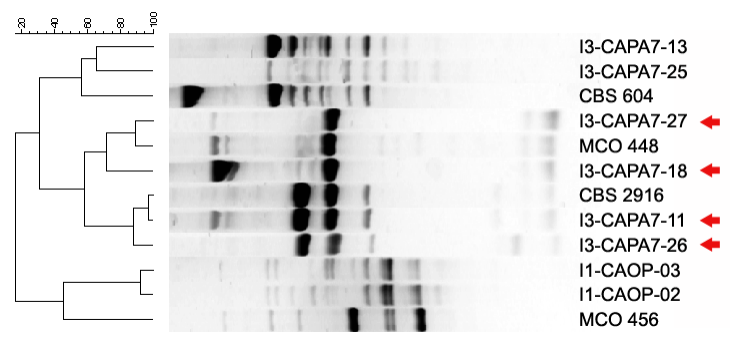
**UPGMA clustering of selected *C. parapsilosis*, *orthopsilosis *and *metapsilosis *strains**. Altogether 4 strains originally identified as *C. parapsilosis *(marked by arrows) showed doubtful profiles in McRAPD. When their fingerprints were compared to fingerprints of selected *C. parapsilosis *(CBS 604), *orthopsilosis *(MCO 456) and *metapsilosis *(CBS 2916 and MCO 448) strains identified and verified earlier, they clustered unquestionably with *C. metapsilosis*.

Clustering of McRAPD data was performed using the UPGMA algorithm performed with similarity coefficients obtained as described in Material and Methods (See additional file 1: Similarity coefficients). This revealed distinct clades of isolates in some of the species, indicating the possibility to recognise distinct genotypes based on McRAPD data (Figure 6, 7, 8, 9, 10, 11, 12, 13 and 14). After correlating these clusters with the appearance of curves visually, thresholds for defining distinct McRAPD genotypes were established in dendrograms empirically (see red vertical lines in Figures 6, 7, 8, 9, 10, 11, 12, 13 and 14). Strains belonging to each genotype are highlighted by different ground tint colors in the dendrograms corresponding with the same colors of curves in accompanying melting curve plots. Those strains not assigned to a specific genotype are not color-coded. When McRAPD data of a particular strain were markedly different compared to data obtained with all the other strains of the same species, RAPD fingerprint of this strain was first inspected and compared with the other strains to verify this discrepancy. In 4 such cases the isolates were originally identified as *C. parapsilosis*, but the RAPD fingerprint unequivocally indicated them as those belonging to the cryptic species *C. metapsilosis*, as demonstrated by comparison to a CBS reference strain and two MCO strains originally classified as groups II and III of *C. parapsilosis *[11,12] and later confirmed to belong to newly recognised species *C. orthopsilosis *and *C. metapsilosis*, respectively [13,14] (Figure 15). Because *C. orthopsilosis *and *C. metapsilosis *cannot be easily differentiated from *C. parapsilosis *using conventional phenotypic identification techniques or using the ID 32C commercial set of assimilation tests (bioMérieux, Marcy l'Etoile, France), the result of McRAPD and RAPD identification cannot be considered as discrepant from the result of conventional phenotyping techniques. In the other cases of doubtful profiles (n = 12), McRAPD either suggested discrepant species identification result or did not suggest any identification. In such cases, the conventional phenotypic species identification was further verified using the ID 32C. Results of this verification are summarized in Table 1. In all cases where McRAPD suggested discrepant identification, further supported by detailed inspection of RAPD fingerprint (n = 9), ID 32C identified the strain in accordance with McRAPD. On the contrary, in all cases where McRAPD and RAPD did not suggest any unequivocal identification (n = 3), ID 32C identified the strain in accordance with conventional phenotypic identification techniques. In the latter cases, the McRAPD profile presumably reflects a unique genotype represented by a single isolate among the strains included in our study. If the original species identification was changed in the above mentioned cases, the original strain labelling which includes original species abbreviation did not change, but the change was indicated by an arrow and new abbreviation in all figures concerned, e.g. I3-CAGU3-01 → CAAL.

**Table 1:** Summary of discrepant identification results.

Strain	Phenotypic identification	McRAPD identification	ID 32C identification
I3-CAKR2-35	*Candida krusei*	*Candida parapsilosis*	*Candida parapsilosis*

I3-CATR9-32	*Candida tropicalis*	*Candida parapsilosis*	*Candida parapsilosis*

I3-CATR9-09	*Candida tropicalis*	*Candida albicans*	*Candida albicans*

I3-SACE3-07	*Saccharomyces cerevisiae*	*Candida tropicalis*	*Candida tropicalis*

I3-SACE3-26	*Saccharomyces cerevisiae*	*Candida lusitaniae*	*Candida lusitaniae*

I1-CAGU2-25	*Candida guilliermondii*	*Saccharomyces cerevisiae*	*Saccharomyces cerevisiae*

I1-CAGU2-26	*Candida guilliermondii*	*Candida albicans*	*Candida albicans*

I1-CAGU2-27	*Candida guilliermondii*	?	*Candida guilliermondii*

CCY 29-4-21	*Candida guilliermondii*	*Candida albicans*	*Candida albicans*

I1-CAPE2-35	*Candida pelliculosa*	*Candida krusei*	*Candida krusei*

I1-CAPE2-36	*Candida pelliculosa*	?	*Candida pelliculosa*

CCY 29-6-7	*Candida pelliculosa*	?	*Candida pelliculosa*

To see whether the strain clustering patterns resulting from McRAPD and conventional RAPD are consistent, McRAPD genotypes were color-coded by ground tint colors in the dendrogram of RAPD fingerprints using different color saturation for different genotypes (additional file 2: Dendrogram of RAPD fingerprints). Whereas McRAPD genotypes correlated very well with RAPD clustering in *C. tropicalis*, the correlation was limited in *C. lusitaniae *and no or almost no correlation was observed in *C. albicans*, *C. krusei*, and *S. cerevisiae *(no McRAPD genotypes were delineated in other species). This is mainly because of different data processing in conventional RAPD versus McRAPD. In RAPD, differences in overall amplification efficiency result in differences in intensity of banding patterns. Therefore, it is strongly recommended not to include weak bands into comparison of RAPD fingerprints, because these can appear or disappear in different amplification runs. Also, the relative intensity of strong bands cannot be reliably taken into account for comparison. That is why we used the band-based Jaccard coefficient for processing of RAPD fingerprints, which takes the position of a band into account but neglects its intensity. In contrast, raw fluorescence measured during melting in the McRAPD procedure truly reflects the relative representation of individual RAPD products (bands in electrophoresis) in the sample. Inter-sample and inter-run differences in overall fluorescence of samples are subsequently proportionally equilibrated during numerical normalization of melting data. Then, relative representation of individual RAPD products is reflected in the slope of a normalized curve or in the height of a peak in a derivative curve and this is taken into account during further evaluation.

### McRAPD data can be used for automated species identification

Since McRAPD data are numerical, the possibility of automated processing aimed to provide accurate identification is self-intriguing. We considered two approaches to achieve this objective. Firstly, absolute differences between normalized melting curves can be easily calculated as described in Material and Methods; such calculation can be simply automated. This should allow us to compare the McRAPD profile of an unknown isolate to a set of profiles obtained with previously identified yeast strains, revealing the closest match. Performance of such automated identification is summarized in Table 2. Overall accurate identification rate was 80%, varying between 58.5 and 100% in different species.

**Table 2:** Accurate identification rate achieved with different approaches to interpretation of McRAPD data.

Species	N	Normalized curves	Normalized curves + matching of derivative peaks	Visual matching of derivative plots	Matching of RAPD fingerprints
*Candida albicans*	44	63.6	72.7	**100**	**100**

*Candida glabrata*	41	58.5	82.9	97.6	97.6

*Candida krusei*	39	64.1	82.1	97.4	**100**

*Candida tropicalis*	40	**100.0**	97.5	**100**	**100**

*Saccharomyces cerevisiae*	39	89.7	92.3	**100**	**100**

*Candida parapsilosis*	38	73.7	78.9	**100**	**100**

*Candida lusitaniae*	41	97.6	97.6	**100**	**100**

*Candida guilliermondii*	19	94.7	94.7	94.7	94.7

*Candida pelliculosa*	17	88.2	82.4	82.4-88.2	**100**

*Candida metapsilosis*	4	75.0	**100.0**	**100**	**100**

**All species studied**	**322**	**79.5**	**86.7**	**98.1-98.4**	**99.4**

Normalized curves column stays for accurate identification rate achieved when identification was based on automated determination of the numerically closest match of the examined curve with known strain. Normalized curve + matching of derivative peaks column stays for the same amended by checking for decisive peaks in derivative plot. Visual matching of derivative plots column stays for accurate identification rate achieved when identification was based on simple visual comparison of examined derivative plot with plots of known strains. Accurate identification rate achieved upon evaluation and matching of RAPD fingerprints is shown for reference in the last column. See Results and discussion for details.

Since the peaks observed in a first derivative plot may in some cases represent the overall characteristic shape of a melting curve better, we also tested performance of matching peaks positions for identification purposes as the second possible approach. However, identification of individual melting peaks in a derivative plot and comparison of these results to those characteristic for each species cannot be automated as easily. Therefore, we first evaluated the presence of individual peaks in each species and each genotype. To reduce the amount of processed data and to identify typical positions of peaks in derivative curves, average first derivative curves were first calculated for each species/genotype based on individual derivation values of each strain of the respective species/genotype. Average curves are summarized in additional file 3: Average derivative curves. To establish the relevance of each averaged peak for species/genotype identification, these were subsequently classified into three categories: (i) decisive which occurred in all strains of the respective species/genotype, (ii) characteristic which occurred in 75-99% of strains of the respective species/genotype, and (iii) possible which occurred in less than 75% of strains. Presence of peaks in individual species/genotypes as described above is summarized in Table 3. Unfortunately, when we tested the reading of peaks positioning alone for yeast identification, unequivocal match was impossible in many cases (data not shown).

**Table 3:** Average melting temperatures of peaks in first derivative plots obtained in individual species/genotypes.

Species	Genotype	Decisive peaks	Characteristic peaks	Possible peaks
*Candida albicans*	A	86.0 ± 0.22	82.9 ± 0.32 83.9 ± 0.27 87.6 ± 0.11	80.0 ± 0.44 82.0 ± 0.31
	
	B	84.1 ± 0.10	85.3 ± 0.17 87.6 ± 0.12	79.9 ± 0.06
	
	C	86.0 ± 0.34	82.0 ± 0.18 82.6 ± 0.30 87.6 ± 0.05	79.8 ± 0.36 83.9 ± 0.29

*Candida tropicalis*	A	82.7 ± 0.27	85.0 ± 0.33	
	
	B	78.9 ± 0.24 82.7 ± 0.23 84.8 ± 0.50		

*Candida parapsilosis*		83.0 ± 0.19 86.6 ± 0.11	84.1 ± 0.19	81.9 ± 0.12

*Candida metapsilosis*		81.2 ± 0.37 83.8 ± 0.12		79.5 ± 0.17

*Candida glabrata*		83.7 ± 0.23	82.1 ± 0.26 85.3 ± 0.22	87.1 ± 0.18 89.0 ± 0.36

*Candida krusei*	A	82.8 ± 0.29	78.6 ± 0.19 85.5 ± 0.19 87.6 ± 0.19 89.2 ± 0.12	
	
	B	83.0 ± 0.22	78.6 ± 0.16 85.5 ± 0.18	83.9 ± 0.11
	
	C	82.9 ± 0.25 85.5 ± 0.06 87.7 ± 0.10 89.1 ± 0.21		78.4 ± 0.07

*Candida lusitaniae*	A	85.4 ± 0.17 86.8 ± 0.15 89.1 ± 0.24		80.4 ± 0.28 82.3 ± 0.19
	
	B	85.5 ± 0.10 86.9 ± 0.08		80.4 ± 0.23 81.6 ± 0.19 82.4 ± 0.19
	
	C	80.7 ± 0.13 83.9 ± 0.13 85.7 ± 0.10 87.0 ± 0.09		
	
	D	85.2 ± 0.06		79.0 ± 0.14 82.8 ± 0.15

*Candida guilliermondii*		82.4 ± 0.12 84.7 ± 0.12	85.6 ± 0.11 86.4 ± 0.10	

*Candida pelliculosa*		85.0 ± 0.16	86.0 ± 0.09	83.8 ± 0.19 88.3 ± 0.24 90.2 ± 0.16

*Saccharomyces cerevisiae*	A	85.1 ± 0.09		
	
	B	84.9 ± 0.16		82.8 ± 0.20

Therefore, we combined the two proposed approaches into one two-step approach. In the first step, the closest match was established between the McRAPD data of the unknown sample and a set of all the other McRAPD profiles in an automated way. Then, a derivative plot was checked for the presence of decisive peaks in the second step. When the examined peak was found to fit in the interval of average peak position ± 2 S.D., it was considered as matched to the average peak. If any of the average decisive peaks characteristic for the best matched species was missing in the examined strain, this best match was evaluated as incorrect identification and the second best match was further evaluated. If the automated identification suggested two very close matches with curves of different species, both concordant in decisive peaks with the examined strain, the characteristic peaks were evaluated and interpreted in favor of one of the matches. Performance of this two step approach was generally much better than the first-step approach alone, with overall accurate identification rate of 87%, varying between 72.7 and 100% in different species. Results of the evaluation are summarized in Table 2. Surprisingly, in *C. tropicalis *and *C. pelliculosa*, the two step approach showed lower sensitivity compared to the first-step alone. This indicates that in some cases the process of matching examined peaks with average decisive peaks ± 2 S.D. precludes correct identification. This is most likely because the interval defined by the position of a decisive peak ± 2 S.D. includes only 95% of the isolates of each genotype. Thus, some isolates among the remaining 5% can prove to match a closely positioned decisive peak of a different species, even if they in fact belong to the species originally suggested by first-step automated processing of McRAPD data.

### Computer-aided visual matching of derivative plots shows excellent performance

**Figure 16:**
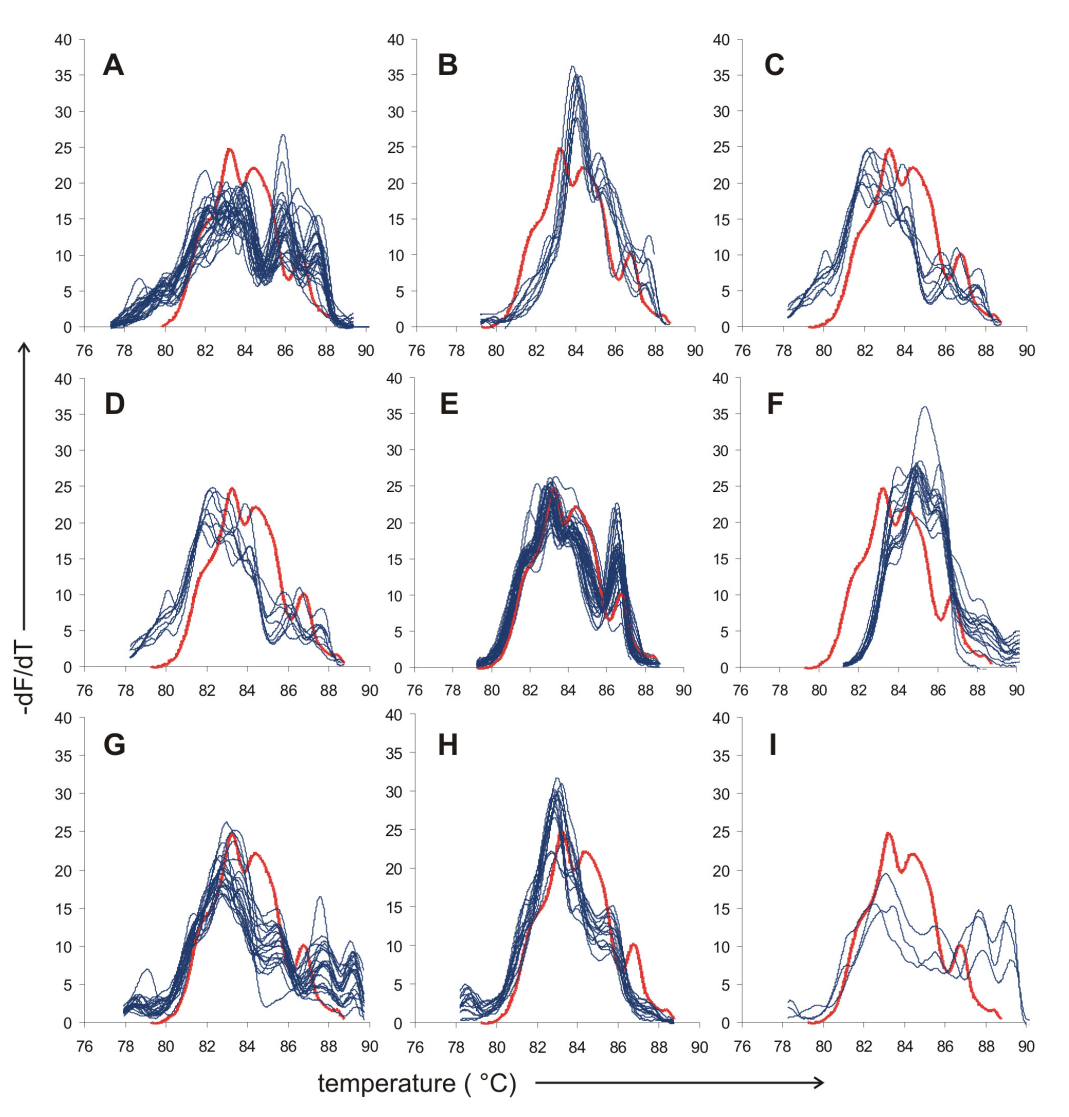
**Visual matching of derivative curves as used for species identification**. Plots of derivative curves obtained with all strains assigned to 9 selected species/genotypes versus the derivative curve obtained with a tested isolate are shown as an example to illustrate the visual matching approach. Curve obtained with the tested isolate is always plotted as a bold red line, whereas curves obtained with strains assigned to a known genotype/species are plotted as thin dark blue lines in separate plots as follows: (A) *C. albicans *genotype A, (B) *C. albicans *genotype B, (C) *C. albicans *genotype C, (D) *C. glabrata*, (E) *C. parapsilosis*, (F) *C. pelliculosa*, (G) *C. krusei *genotype A, (H) *C. krusei *genotype B, (I) *C. krusei *genotype C.

Since the performance of proposed automated identification approach followed by matching the peaks positions has not reached the accuracy of identification based on traditional RAPD fingerprints, we further looked for other ways to best interpret the information present in melting curves. Simple visual inspection of a derivative curve obtained with the examined strain and its comparison to sets of curves obtained with isolates belonging to each clearly delineated species genotype appeared intuitively as the most promising alternative. To achieve this comparison in an easy-to-manage way we developed a simple computer-aided plotting scheme. Using Microsoft Excel 2007 software, plots of all derivative curves assigned to each species/genotype were prepared in separate sheets using thin lines and the curve of a tested isolate was then imported into another sheet and automatically plotted into each of the plots using a bold line. Then, all of the plots of specific species/genotypes including the bold curve of the tested isolate were inspected visually and the best match was evaluated based on subjective judgment (see Figure 16 for an example). This evaluation was performed independently by two people in a blinded fashion, i.e. the evaluating person did not know the identity of any of the tested curves and the curves were selected in a random order for evaluation to avoid any bias. Later, a third person evaluated the accuracy of this subjective visual identification using a key generated during randomization. Altogether, 316 and 317 of 322 isolates were identified correctly, achieving excellent accuracy of 98.1-98.4% (for results in individual species see Table 2). In other words, 6 strains were misidentified by one evaluator and 5 strains by the other, where the 6 strains misidentified by one evaluator included the 5 strains misidentified by the other. This concordance indicates clearly that this failure was not caused by subjective error, but rather by lack of typical properties in the misidentified melting profiles. Closer inspection of the misidentified strains showed that they included one strain which showed a completely unique fingerprint and therefore was not identified by traditional RAPD fingerprinting, and other 2 strains which showed less characteristic fingerprints, albeit it was possible to identify them using traditional RAPD fingerprinting.

## Discussion

Our results show that McRAPD offers a promising alternative to conventional phenotypic identification techniques. Surprisingly, simple visual inspection of derivative plots performed best among the approaches tested for interpretation of mere numerical McRAPD data. Its performance almost matched the performance of traditional RAPD fingerprinting. Compared to the automated processing developed and tested by ourselves, the time costs of simple visual evaluation were roughly equal when using a pre-made computer-aided plotting scheme. However, with a broader spectrum of yeast species and expanding database of McRAPD results, simple visual examination can become more time demanding and cumbersome. Therefore, it may be advantageous to test for a threshold score in automated matching which can guarantee flawless identification in the future. Then, the visual matching could be reserved for isolates failing to reach this score in automated matching. When looking at the accuracy of identification obtained in this study, this should be regarded critically in the light of the fact that all of the evaluations were based on an artificially assembled set of strains. However, because this set comprised almost 95% of species typically isolated from clinical samples, real performance in routine settings should not differ too much. An ongoing prospective study being performed by ourselves should prove this assumption.

When evaluating the future potential of McRAPD, we should first consider the main advantages and disadvantages of the RAPD technique itself. It is well-known that RAPD is highly sensitive not only to minor inter-strain differences, but also to minor differences in experimental conditions, which can result in different profiles, compromising intra- and interlaboratory reproducibility. There are many factors that can influence the appearance or disappearance of bands, including Mg^2+ ^concentration, primer/template concentration ratio, Taq polymerase concentration and source, the model of thermal cycler etc. [15-18]. Since we aimed to use RAPD/McRAPD primarily not for strain typing but for species identification purposes, we optimised the amplification conditions in favour of low interstrain variability. This efficiently prevented problems with intralaboratory reproducibility, as clearly demonstrated in Figure 4 and discussed above. Of course, some problems may occur with interlaboratory reproducibility, mainly when using a different model of thermal cycler or a different Taq polymerase. However, machines using air heating and cooling of samples which secure rapid and accurate ramping and rapid cycling should be able to minimise this problem, as documented in our study using glass capillaries and RapidCycler 2 machine. Also, commercial polymerases with guaranteed performance are available globally. Therefore, we believe that these drawbacks can be at least compensated or even outweighed by the advantages of McRAPD. Firstly, RAPD itself is very easy and economical to perform, which makes it the second most widely used genotyping technique in yeast microbiology as illustrated by 92 citations in PubMed for "(RAPD OR AP-PCR) AND typing AND yeast" versus 139 for RFLP, 40 for PFGE, 30 for MLST, and 9 for AFLP. In addition, its usefulness for yeast species identification was documented by several groups independently [7,19-23]. To the best of our knowledge, all of the other genotyping techniques are more laborious and less economical for the purpose of species identification. If there is a technology for melting analysis available, McRAPD is even easier and more economical to perform than RAPD, because it does not require gel electrophoresis. However, omitting the electrophoresis also means that a visual check of proper amplification is not possible. This can question the reliability of McRAPD results, because as in any PCR, RAPD amplification can also occur in negative controls, for reasons well documented earlier [24,25]. Then, performance of DNA extraction can be another source of inadequate McRAPD performance, because it may not recover enough template DNA of adequate quality for amplification, opening the door for false RAPD amplification. However, this risk can be significantly reduced by applying the criterion of the relative value of fluorescence reaching a critical threshold, as used in this study. When a real-time cycler is used for amplification, a monitoring of fluorescence during McRAPD also allows for controlling the reliability of McRAPD data, because slow amplification of a specific sample as compared to standard samples clearly indicates improper performance, most likely because of the inadequate quality of template DNA. In this case, real-time amplification should reveal the failure of McRAPD even better than gel electrophoresis which can only demonstrate the end-point result of PCR amplification.

When comparing the McRAPD performance to its alternatives available in routine laboratories, we have clearly demonstrated that it performs better than conventional phenotypic identification techniques which are in addition much more time-consuming. In this study we do not provide any direct and extensive comparison to other approaches, except the limited comparison to the commercial assimilation set ID 32C. Among the 20 strains examined both by McRAPD and ID 32C, the results were concordant in 9 cases and McRAPD was superior to ID 32C in 4 strains of *C. metapsilosis*, whereas ID 32C was superior to McRAPD in 3 strains where McRAPD failed to suggest any identification. As already noted above, these cases of unmatched McRAPD profiles presumably represent a unique genotype represented by a single isolate among the strains included in our study. The database of standard McRAPD results is now very limited compared to ID 32C but can be expected to grow in future. This should help to resolve such cases. In addition, if McRAPD does not suggest any match or if there are any doubts about the match suggested, there is always an option of subsequent gel electrophoresis of the same sample that reveals a classical fingerprint. As clearly demonstrated in a dendrogram based on RAPD fingerprints of all strains included in the study (see additional file 2: Dendrogram of RAPD fingerprints), analysis of RAPD fingerprinting patterns always provided accurate identification except for 2 strains showing quite unique fingerprints (*C. glabrata *CCY 26-20-21 and *C. guilliermondii *I1-CAGU2-27, marked by arrows in the additional file 2: Dendrogram of RAPD fingerprints). Importantly, RAPD also identified correctly 2 of the 3 strains where McRAPD failed to suggest any identification. It should also be noted, that our study was performed with one single primer only. This primer showed very good performance with uniform melting profiles in most species, but also less uniform profiles in few other species. It can hardly be expected that one single primer can cover McRAPD identification of all medically important yeast species without problems. Thus, future studies may improve the performance of the McRAPD approach also by testing more primer systems and suggesting the best mixes. This was out of the scope of this study.

When comparing the routine processing of samples in McRAPD and ID 32C, both require pure culture of the respective yeast strain. Whereas ID 32C requires 1-3 colonies to achieve 2 ml of suspension medium showing turbidity of McFarland 2, sampling of a small fraction of one colony is enough for McRAPD as described in Materials and Methods. Concerning the time needed to achieve identification, McRAPD can be finished within 3.5 hours if simple DNA extraction is performed and a real-time cycler with high-resolution melting analysis option is available, whereas ID 32C can be read only after 24-48 hours reliably, as recommended by the manufacturer. Of course, both techniques can fail, e.g. with an unrecognised mixed culture. In such case, McRAPD repetition is completed within a few hours on the next day, whereas repeating ID 32C needs further 2 days. Concerning the labour time, McRAPD requires about 1.5 hours to process 10-20 samples, whereas ID 32C needs about 5 min to prepare a set for incubation and 1-3 min to evaluate the results per sample, i.e. about 1-2 hours to process 10-20 samples. Comparison of costs cannot be accomplished easily. Whereas McRAPD requires special and expensive instrumentation, ID 32C can be used in any cultivation laboratory without any special equipment. On the other hand, real-time PCR machines with high resolution melting option needed for McRAPD can be expected to gradually become a general must, at least in advanced routine microbiological laboratories. When comparing operation costs of both procedures, our experience shows that McRAPD can be quite competitive compared to ID 32C, however, market prices of materials and sets are always subject to change.

Thus, it should be fair to say that both approaches are roughly comparable, McRAPD being more rapid with a potential for future improvements. Since ID 32C offers the most extensive set of assimilation tests among commercially available yeast identification systems, it can be expected that other phenotyping approaches will show inferior performance. Thus, the need of special instrumentation and skills should be the only obstacle for general acceptance of McRAPD in routine diagnostic laboratories. Generally speaking, those laboratories being able to adopt McRAPD will be also able to adopt other genotyping techniques. Then, such techniques, Multi Locus Sequence Typing (MLST) in particular, should be the main competitors of McRAPD. Although MLST is more demanding concerning instrumentation, skills and labour, it has the advantage of unmatched interlaboratory reproducibility, enabling global epidemiology. However, it can hardly be expected that MLST can present an economically affordable alternative for routine identification and prospective epidemiological surveillance in near future. It can rather be expected that its use will be limited to retrospective epidemiological studies. Thus, McRAPD offers a promising choice for routine identification of pathogenic yeast species; in case of failure, it could be supplemented by other techniques, the best of which appears to be single-locus sequencing in our opinion.

## Conclusions

1. Crude colony lysates provide an economical, rapid and reliable alternative to elaborate DNA extraction techniques for the purposes of McRAPD when performed by skilled personnel.

2. Our optimized McRAPD protocol shows excellent intralaboratory reproducibility and is able to delineate specific genotypes in some of the species studied.

3. Computer-aided visual matching of first derivative plots shows best performance among the approaches tested for interpretation of mere numerical McRAPD data. Its performance almost matched the performance of traditional RAPD fingerprinting and was comparable to the performance of the ID32C commercial system.

4. We believe that because of its advantages over conventional phenotypic identification approaches and competitive costs McRAPD can find its place in routine identification of medically important yeasts in advanced diagnostic laboratories being able to adopt the technique. It can also serve as a broad-range high-throughput technique for crude epidemiological surveillance.

## Methods

### Yeast strains

The 9 yeast species most frequently isolated from clinical samples in our settings, namely representing 94.3% of yeast species isolated from patient samples at our department, were included into the study. Among these, 7 more common species, i.e. *Candida albicans *(56.2%), *C. glabrata *(12.6%), *C. krusei *(8%), *C. tropicalis *(7.7%), *Saccharomyces cerevisiae *(3.1%), *C. parapsilosis *(2.5%), and *C. lusitaniae *(2%) were represented by at least 35 isolates each, whereas the less frequently isolated species *C. guilliermondii *(1.3%) and *C. pelliculosa *(1%) were represented by at least 15 isolates each. A few isolates of *C. orthopsilosis *and *C. metapsilosis *were also included into the study later, when described as cryptic species of *C. parapsilosis *[13]. See also additional file 4: Listing of clinical isolates and reference strains included in this study. The strains were stored in 20% BBL Skim Milk Powder supplemented with glycerol (BD, Franklin Lakes, New Jersey, USA) at -70°C until used.

### Phenotypic identification

All of the isolates were identified using conventional phenotypic identification techniques, i.e. evaluation of micromorphology on rice agar and evaluation of biochemical properties using in-house prepared assimilation and fermentation tests [26] followed by interpretation using the identification key according to Fragner [27]. Selected isolates were also identified using the ID 32C commercial set (bioMérieux, Marcy l'Etoile, France) in accordance with manufacturer's instructions.

### DNA extraction

Crude colony lysates described earlier as suitable for amplification were prepared by simple toothpick technique [7]. Briefly, a part of colony grown on SGA plate was picked up by a micropipette tip at latest one day after inoculation and transferred into 5 μl of freshly prepared lysing solution (1 M sorbitol, 5 mM MgCl_2_, 2 mM dithiothreitol, 12 U of Zymolyase, all from Sigma-Aldrich, St. Louis, Missouri, USA). The mixture was incubated for 30 min at 37°C and centrifuged (10,000 g for 5 min). The supernatant was transferred into a new tube, diluted with TE buffer to 300 μl and stored at -20°C until used. For comparison and reference, YeaStar Genomic DNA Kit (Zymo Research, Orange, California, USA) was also used for DNA extraction in selected strains following manufacturer's recommendations. Briefly, 1 ml of yeast submerged culture (approx. 1.5 × 10^7 ^cells) grown in YPG (1% of each yeast extract, peptone and glucose) in an Erlenmeyer flask shaken at 30°C was spun down and the pellet was subjected to enzyme lysis in 120 μl of YD Digestion Buffer (containing RNase A and Zymolyase) for 1 hour at 37°C. Then, 120 μl of YD Lysis Buffer and 250 μl of chloroform were added, mixed and spun down again. The aqueous supernatant was then loaded onto a fast spin-column, spun down, and the impurities were washed away using DNA Wash Buffer. Finally, DNA was eluted by 60 μl of water.

### McRAPD procedure

PCR reaction was performed in a glass capillary in a total volume of 10 μl consisting of 0.5 μM primer ACGGGCCAGT [21], 10 mM Tris-HCl (pH 8.8), 50 mM KCl, 0.1% Triton X-100, 2 mM MgCl_2_, 200 μM of each dNTP, 2.5 U of Taq polymerase Unis (Top-Bio, Prague, Czech Republic), 250 μg/ml BSA and LCGreen dye at 1× concentration (Idaho Technology Inc., Salt Lake City, Utah, USA). Either 1 μl of crude colony lysate or 1 μl of DNA extracted using the YeaStar Genomic DNA Kit was added into the reaction. Amplification was performed in a Rapid Cycler 2 apparatus (Idaho Technology Inc., Salt Lake City, Utah, USA) applying an empirically optimized protocol of initial denaturation at 95°C, 5 min, followed by 45 cycles of denaturation at 95°C for 5 s, annealing at 48°C for 10 s, and extension at 72°C for 40 s, with ramping 1°C/s, followed by final extension at 72°C for 5 min.

### Analysis of McRAPD data

RAPD amplicons were subjected to melting analysis on a high-resolution melting instrument HR-1 (Idaho Technology Inc., Salt Lake City, Utah, USA). The samples were heated at ramping rate of 0.3°C/s with acquisition of fluorescence data ranging from 75 to 95°C. Results were analysed using the HR-1 melt analysis software. Relative fluorescence was first plotted versus temperature and fluorescence intensity values were normalized as recommended by the manufacturer. For this purpose, temperature ranges preceding and following the melting domain were optimized empirically to result in reproducible normalized melting curves in all of the yeast species examined. The optimized intervals for normalization were 75.5-77.5°C and 91.5-93.5°C, respectively. A simple procedure for comparison of normalized melting profiles was developed by us. Briefly, differences in McRAPD data of a pair of isolates were calculated by subtracting their normalized fluorescence values measured at each temperature point during melting analysis. Then, the sum of these subtracted values represented absolute numerical distance between the pair of isolates, i.e.:

where

*AD*_1,2 _was absolute distance between isolates No. 1 and 2

*f*_1_(*t*) was normalized fluorescence of isolate No. 1 measured at temperature *t*

*f*_2_(*t*)was normalized fluorescence of isolate No. 2 measured at temperature *t*

After the absolute distance was established in all pairs (combinations) of isolates, the relative distance 1.0 was assigned to the highest absolute value obtained in the most dissimilar (numerically distant) pair of isolates, abbreviated as *AD*_max_. Relative distance values for the remaining pairs of isolates were calculated as a fraction of the highest absolute value, i.e.:

A matrix of relative distances was assembled for the isolates included into each comparison. Then, the matrix of relative distances was used to calculate tree data for a cladogram using the UPGMA method and Phylip software [28,29]. PhyloDraw 0.8 software [30,31] was used for cladogram construction.

For additional analysis, plots of the first negative derivation of fluorescence depending on temperature were also prepared based on melting data normalized previously. To delineate the melting peaks better, smoothing of data was performed using the HR-1 analysis software as recommended by the manufacturer. In some cases this smoothing resulted in truncation of the left and/or right end of the derivative curve. This process was carefully observed to prevent any loss of potentially discriminatory peaks at both ends of the derivative curves. To prevent excessive simplification and loss of informative data, smoothing was performed only if it undoubtedly resulted in a distinct amelioration of peaks' discrimination.

### Electrophoresis and analysis of banding patterns

After melting analysis was performed, each sample was also subjected to gel electrophoresis in 2% agarose gel at 5 V/cm for 3 hours. The gels were stained by ethidium bromide added into them during preparation at the final concentration of 1 μg/ml and resulting banding patterns were photographed. Comparison of fingerprints was performed using GelCompar II software (Applied Maths, Sint-Martens-Latem, Belgium) applying the Jaccard coefficient at 1.5% positioning tolerance. Dendrograms were constructed using the UPGMA algorithm.

## Authors' contributions

JT performed most of the DNA extractions and McRAPD amplification, processed the acquired data, performed statistical analysis and drafted the paper. PP developed a software tool to facilitate comparison of normalized McRAPD data. LR participated in DNA extractions and McRAPD amplification. PH and DK performed conventional phenotypic identification of yeast species as well as ID 32C identification of selected strains and revised the paper critically. VR conceived and designed the study, developed the concept of automated processing of McRAPD data, participated in drafting the paper, revised it critically and gave final approval of the version to be published. All authors read and approved the final manuscript.

## Supplementary Material

Additional file 1 **Similarity coefficients**. Listing of similarity coefficients obtained upon automated comparison of normalized melting curves within each species. Click here for file

Additional file 2 **Dendrogram of RAPD fingerprints**. Dendrogram based on RAPD fingerprints of all strains included in the study. Analysis of RAPD fingerprinting patterns always provided accurate identification except for 2 strains showing quite unique fingerprints (marked by arrows). For comparison of strain clustering between conventional RAPD and McRAPD, the strains of different species are color-coded by ground tint colors and their specific McRAPD genotypes are indicated by different saturation of colors. In case a strain was not assigned to a specific McRAPD genotype, it is not color-coded. Click here for file

Additional file 3 **Average derivative curves**. Plots of average McRAPD first negative derivative curves of species and genotypes included in the study. Click here for file

Additional file 4 **Listing of clinical isolates and reference strains included in this study**. Click here for file
