# Supplementary figures and images for: Performance of optimized McRAPD in identification of 9 yeast species frequently isolated from patient samples: potential for automation
Source: BMC Microbiol. 2009 Nov 10;9:234. doi: 10.1186/1471-2180-9-234 (PMC2779194; doi:10.1186/1471-2180-9-234)

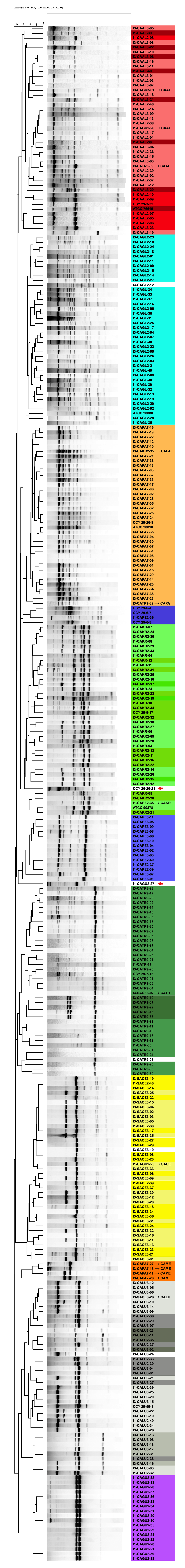

Supplement: Additional file 2 — Dendrogram of RAPD fingerprints. Dendrogram based on RAPD fingerprints of all strains included in the study. Analysis of RAPD fingerprinting patterns always provided accurate identification except for 2 strains showing quite unique fingerprints (marked by arrows). For comparison of strain clustering between conventional RAPD and McRAPD, the strains of different species are color-coded by ground tint colors and their specific McRAPD genotypes are indicated by different saturation of colors. In case a strain was not assigned to a specific McRAPD genotype, it is not color-coded. [file 1471-2180-9-234-S2.png]
