# Supplementary material for: Performance of optimized McRAPD in identification of 9 yeast species frequently isolated from patient samples: potential for automation
Source: BMC Microbiol. 2009 Nov 10;9:234. doi: 10.1186/1471-2180-9-234 (PMC2779194; doi:10.1186/1471-2180-9-234)
Supplement: Additional file 4 — Listing of clinical isolates and reference strains included in this study. [file 1471-2180-9-234-S4.pdf]

Additional file 4.
Clinical isolates and reference strains included in this study.

Species	Strain	Source
Candida albicans	I1-CAAL2-36	Sputum
	I1-CAAL2-05	Throat
	I1-CAAL2-37	Nail
	I1-CAAL2-38	Stool
	I1-CAAL2-39	Urine
	I1-CAAL2-06	BAL fluid
	I1-CAAL2-07	Stool
	I1-CAAL2-40	Cannula
	I3-CAAL3-01	Skin
	I1-CAAL-39	Puncture
	I3-CAAL3-02	Gastric secretion
	I1-CAAL2-09	Cannula
	I3-CAAL3-03	Catheter
	I3-CAAL3-04	Sputum
	I3-CAAL3-05	Stool
	I1-CAAL-38	Gastric secretion
	I1-CAAL2-08	Throat
	I1-CAAL-40	Cannula
	I1-CAAL2-01	Stool
	I3-CAAL3-06	Bile
	I3-CAAL3-07	Cannula
	I3-CAAL3-08	Vagina
	I3-CAAL3-09	Drain
	I3-CAAL3-10	Vagina
	I1-CAAL2-03	Buccal cavity
	I3-CAAL3-11	Ear
	I3-CAAL3-12	Bile
	I3-CAAL3-13	Stool
	I1-CAAL2-10	Cannula
	I3-CAAL3-14	Blood culture
	I3-CAAL3-15	Blood culture
	I3-CAAL3-16	Blood culture
	I3-CAAL3-17	Blood culture
	I3-CAAL3-18*	Cerebrospinal fluid

	I3-CAAL3-19*	Blood culture
	I3-CAAL3-20*	Urine
	I3-CAAL3-21*	Urine
	I3-CAAL3-22*	Urine
	I3-CAAL3-23*	Urine
	CCY 29-3-32	Reference strain
	ATCC 76615	Reference strain
Candida tropicalis	I1-CATR-17	Wound
	I1-CATR-36	Buccal cavity
	I3-CATR8-28	Urine
	I3-CATR9-01	Sputum
	I3-CATR9-02	Ear
	I3-CATR9-03	Bile
	I3-CATR9-04	Catheter
	I3-CATR9-05	Secretion
	I3-CATR9-06	Aspirate
	I3-CATR9-07	Punction
	I3-CATR9-08	Stool
	I3-CATR9-09	Vagine
	I3-CATR9-10	Buccal cavity
	I3-CATR9-10	Buccal cavity
	I3-CATR9-11	Nail
	I3-CATR9-12	Buccal cavity
	I3-CATR9-13	Throat
	I3-CATR9-14	Bile
	I3-CATR9-15	Nail
	I3-CATR9-16	Buccal cavity
	I3-CATR9-17	Buccal cavity
	I3-CATR9-18	Throat
	I3-CATR9-19	Throat
	I3-CATR9-20	Nail
	I3-CATR9-21	Urine
	I3-CATR9-22	Cannula
	I3-CATR9-23	Cannula
	I3-CATR9-24	Cannula
	I3-CATR9-25	Rectum
	I3-CATR9-26	Throat
	I3-CATR9-27	Stool

	I3-CATR9-28	Cannula
	I3-CATR9-29	Urine
	I3-CATR9-30	Stool
	I3-CATR9-31	Blood culture
	I3-CATR9-32	Blood culture
	I3-CATR9-33	Blood culture
	I3-CATR9-34*	Catheter
	I3-CATR9-35*	Urine
	I3-CATR9-36*	Urine
	I3-CATR9-37*	Urine
	CCY 29-7-12	Reference strain
Candida krusei	I1-CAKR-03	Rectum
	I1-CAKR-04	BAL fluid
	I1-CAKR-05	Sinus swab
	I1-CAKR-06	BAL fluid
	I1-CAKR-07	Throat
	I1-CAKR-08	Sputum
	I1-CAKR-10	Stool
	I1-CAKR-11	Vagina
	I1-CAKR-12	Throat
	I1-CAKR-24	Throat
	I3-CAKR2-09	Throat
	I3-CAKR2-10	BAL fluid
	I3-CAKR2-11	Urine
	I3-CAKR2-12	Sputum
	I3-CAKR2-13	Vagina
	I3-CAKR2-14	Sputum
	I3-CAKR2-15	Sputum
	I3-CAKR2-16	Sputum
	I3-CAKR2-17	Throat
	I3-CAKR2-18	Throat
	I3-CAKR2-19	Wound
	I3-CAKR2-20	Wound
	I3-CAKR2-21	Sputum
	I3-CAKR2-22	Urine
	I3-CAKR2-23	Vagina
	I3-CAKR2-24	Sputum
	I3-CAKR2-25	Throat

	I3-CAKR2-26	Throat
	I3-CAKR2-27	Sputum
	I3-CAKR2-28	Urine
	I3-CAKR2-29	Urine
	I3-CAKR2-30	Bile
	I3-CAKR2-31	Blood culture
	I3-CAKR2-32	Blood culture
	I3-CAKR2-33	Blood culture
	I3-CAKR2-34	Blood culture
	I3-CAKR2-35	Blood culture
	ATCC 90878	Reference strain
	CCY 29-9-17	Reference strain
Candida parapsilosis	I3-CAPA7-01	Cannula
	I3-CAPA7-02	Vagina
	I3-CAPA7-03	Ear
	I3-CAPA7-04	Urine
	I3-CAPA7-05	Ear
	I3-CAPA7-06	Urine
	I3-CAPA7-07	Vagina
	I3-CAPA7-08	Cannula
	I3-CAPA7-09	Throat
	I3-CAPA7-10	Wound
	I3-CAPA7-11	Meninges
	I3-CAPA7-12	Conjunctiva
	I3-CAPA7-13	Conjunctiva
	I3-CAPA7-14	Wound
	I3-CAPA7-15	Nail
	I3-CAPA7-16	Nail
	I3-CAPA7-17	Nail
	I3-CAPA7-18	Nail
	I3-CAPA7-19	Nail
	I3-CAPA7-20	Throat
	I3-CAPA7-21	Ear
	I3-CAPA7-22	Ear
	I3-CAPA7-23	Urine
	I3-CAPA7-24	Cannula
	I3-CAPA7-25	Urine
	I3-CAPA7-26	Skin

	I3-CAPA7-27	Ear
	I3-CAPA7-28	Cannula
	I3-CAPA7-29	Cannula
	I3-CAPA7-30	Cannula
	I3-CAPA7-31	Sputum
	I3-CAPA7-32	Dialysis liquid
	I3-CAPA7-33	Nail
	I3-CAPA7-34	Nail
	I3-CAPA7-35	Blood culture
	I3-CAPA7-36	Blood culture
	I3-CAPA7-37	Blood culture
	I3-CAPA7-38	Blood culture
	ATCC 90018	Reference strain
	CCY 29-20-8	Reference strain
	CBS 604	Reference strain
Candida glabrata	I1-CAGL-30	Stool
	I1-CAGL-31	Urine
	I1-CAGL-32	Urine
	I1-CAGL-33	Sputum
	I1-CAGL-34	Perianal swab
	I1-CAGL-35	Rectal swab
	I1-CAGL-36	Vagina
	I1-CAGL-37	Cannula
	I1-CAGL-38	Sputum
	I1-CAGL-39	Sputum
	I1-CAGL-40	Throat
	I3-CAGL2-01	Sputum
	I3-CAGL2-02	Urine
	I3-CAGL2-03	Throat
	I3-CAGL2-04	Buccal cavity
	I3-CAGL2-05	BAL fluid
	I3-CAGL2-06	Urine catheter
	I3-CAGL2-07	Wound
	I3-CAGL2-08	BAL fluid
	I3-CAGL2-09	Wound
	I3-CAGL2-10	Perianal swab
	I3-CAGL2-11	Vagina
	I3-CAGL2-12	BAL fluid

	I3-CAGL2-13	Cannula
	I3-CAGL2-14	Vagina
	I3-CAGL2-15	Cannula
	I3-CAGL2-16	Vagina
	I3-CAGL2-17	Sputum
	I3-CAGL2-18	Throat
	I3-CAGL2-19	Sinus catheter
	I3-CAGL2-20	Cannula
	I3-CAGL2-21	Bile
	I3-CAGL2-22	Vagina
	I3-CAGL2-23	Secretion
	I3-CAGL2-24	Cannula
	I3-CAGL2-25	Blood culture
	I3-CAGL2-26	Blood culture
	I3-CAGL2-27	Blood culture
	I3-CAGL2-28	Blood culture
	ATCC 90080	Reference strain
	CCY 26-20-21	Reference strain
Candida lusitaniae	I1-CALU2-29	Skin
	I1-CALU2-30	Throat
	I1-CALU2-31	Throat
	I1-CALU2-32	Catheter
	I1-CALU2-33	Sputum
	I1-CALU2-34	Stool
	I1-CALU2-35	Urine
	I1-CALU2-36	Throat
	I1-CALU2-37	Urine
	I1-CALU2-38	Urine
	I1-CALU2-39	Urine
	I1-CALU2-40	Wound secretion
	I3-CALU3-01	Catheter
	I3-CALU3-02	Sputum
	I3-CALU3-03	Blood culture
	I3-CALU3-04	Nail
	I3-CALU3-05	Urine
	I3-CALU3-06	Ear
	I3-CALU3-07	Throat
	I3-CALU3-08	Urine

	I3-CALU3-09	Wound
	I3-CALU3-10	Cannula
	I3-CALU3-11	Throat
	I3-CALU3-12	Urine
	I3-CALU3-13	Sputum
	I3-CALU3-14	Throat
	I3-CALU3-15	Urine
	I3-CALU3-16	Throat
	I3-CALU3-17	Throat
	I3-CALU3-18	Nail
	I3-CALU3-19	Throat
	I3-CALU3-20	Throat
	I3-CALU3-21	Urine
	I3-CALU3-22	Throat
	I3-CALU3-23	Throat
	I3-CALU3-24	Cannula
	I3-CALU3-25	Urine
	I3-CALU3-26	Urine
	I3-CALU3-27	Stool
	I1-CALU-33	Cannula
	CCY 29-59-1	Reference strain
Candida pelliculosa	I1-CAPE2-35	Throat
	I1-CAPE2-36	Urine
	I1-CAPE2-37	Urine
	I1-CAPE2-38	Throat
	I1-CAPE2-39	Sputum
	I1-CAPE2-40	Buccal cavity
	I3-CAPE3-01	Urine
	I3-CAPE3-02	Throat
	I3-CAPE3-03	Urine
	I3-CAPE3-04	Throat
	I3-CAPE3-05	Urine
	I3-CAPE3-06	Urine
	I3-CAPE3-07	Stool
	I3-CAPE3-08	Cannula
	I3-CAPE3-09	Throat
	I3-CAPE3-10	Urine
	I3-CAPE3-11	Throat

	CCY 29-6-4	Reference strain
	CCY 29-6-7	Reference strain
	CCY 29-6-8	Reference strain
Candida guilliermondii	I1-CAGU-22	Cannula
	I1-CAGU2-20	Urine
	I1-CAGU2-21	Ear
	I1-CAGU2-22	Throat
	I1-CAGU2-23	Nail
	I1-CAGU2-24	Nail
	I1-CAGU2-25	Sputum
	I1-CAGU2-26	Nail
	I1-CAGU2-27	Nail
	I1-CAGU2-28	Wound swab
	I1-CAGU2-29	Blood culture
	I1-CAGU2-30	Urine
	I1-CAGU2-31	Skin
	I1-CAGU2-32	Nail
	I1-CAGU2-33	Throat/nose
	I1-CAGU2-34	Ear
	I1-CAGU2-35	Skin
	I1-CAGU2-36	Skin
	I1-CAGU2-37	Buccal cavity
	I1-CAGU2-38	Wound swab
	I1-CAGU2-39	Sputum
	I1-CAGU2-40	Throat
	CCY 29-4-21	Reference strain
Saccharomyces cerevisiae	I1-SACE2-38	Throat
	I1-SACE2-39	Vagina
	I1-SACE2-40	Perianal swab
	I3-SACE3-01	Vagina
	I3-SACE3-02	Skin
	I3-SACE3-03	Stool
	I3-SACE3-04	Throat
	I3-SACE3-05	Tongue
	I3-SACE3-06	Throat
	I3-SACE3-07	Stool
	I3-SACE3-08	Perianal swab
	I3-SACE3-09	Vagina

	I3-SACE3-10	Perianal swab
	I3-SACE3-11	Stool
	I3-SACE3-12	Stool
	I3-SACE3-13	Throat
	I3-SACE3-14	Abscess
	I3-SACE3-15	Throat
	I3-SACE3-16	Vagina
	I3-SACE3-17	Vagina
	I3-SACE3-18	Sputum
	I3-SACE3-19	Throat
	I3-SACE3-20	Buccal cavity
	I3-SACE3-21	Sputum
	I3-SACE3-22	Vagina
	I3-SACE3-23	Sputum
	I3-SACE3-24	Throat
	I3-SACE3-25	Nail
	I3-SACE3-26	Vagina
	I3-SACE3-27	Throat
	I3-SACE3-28	Stool
	I3-SACE3-29	Skin
	I3-SACE3-30	Sputum
	I3-SACE3-31	Throat
	I3-SACE3-32	Vagina
	I3-SACE3-33	Vagina
	I3-SACE3-34	Wound
	I3-SACE3-35	Secretion
	I3-SACE3-36	Buccal cavity
	I3-SACE3-37	Vagina
Candida orthopsilosis	MCO 456[+]	Blood culture
	I1-CAOP-02	Blood culture
	I1-CAOP-03	Blood culture
Candida metapsilosis	MCO 448[+]	Skin
	CBS 2916	Reference strain

*gifted by Dr. Mine Yücesoy
[+] gifted by Dr. Jozef Nosek
